# Efficacy of Early Health Intervention Programs on Adverse Effects of Chemotherapy Among Women With Breast Cancer: A Randomized Controlled Trial

**DOI:** 10.7759/cureus.63604

**Published:** 2024-07-01

**Authors:** Beena Koshy, Seetha Lakshmi Avudaiappan, Aravindh S Anand

**Affiliations:** 1 Nursing, Government Medical College, Thiruvananthapuram, Thiruvananthapuram, IND; 2 Nursing, Sri Ramachandra Institute of Higher Education and Research, Chennai, IND; 3 Nursing Foundation, Sri Ramachandra Institute of Higher Education and Research, Chennai, IND; 4 Radiation Oncology, Government Medical College, Thiruvananthapuram, Thiruvananthapuram, IND

**Keywords:** adverse effects, chemotherapy, breast cancer, progressive muscle relaxation, health education

## Abstract

Aim

Breast cancer is the most frequently diagnosed cancer and the primary cause of cancer-related mortality among women. Advances in medical science have led to chemotherapy drugs that significantly reduce cancer mortality and increase patient’s life expectancy. However, the systemic nature of chemotherapy leads to a wide range of physical and psychosocial challenges. Chemotherapy is usually given on an outpatient basis and hence patients have to manage treatment-related symptoms at home. This study aimed to evaluate the efficacy of early health intervention programs, specifically health education and progressive muscle relaxation, in managing the adverse effects of chemotherapy among women with breast cancer.

Methods

A randomized controlled trial was carried out at the chemotherapy unit of a tertiary care hospital in Thiruvananthapuram, Kerala, India. The research involved 340 female breast cancer patients receiving their initial chemotherapy cycle, divided equally into an experimental group and a control group. Patients in the intervention group received an early health intervention program on the day of their first chemotherapy cycle. These interventions included a 40-minute session comprising health education to manage the adverse effects of chemotherapy at home and a demonstration of progressive muscle relaxation techniques, which must be practiced by the patients two times daily till the end of chemotherapy. Participants in the control group received routine care from the hospital. The primary outcome variable was the adverse effects of chemotherapy. Sociodemographic and clinical information were collected using a structured questionnaire. The severity of adverse effects was assessed using the Common Terminology Criteria for Adverse Events, version 3 (CTCAE v3).

Result

The average age of participants was 54.7 ± 9.7 years in the control group and 52.4 ± 9 years in the experimental group. The majority in both groups had invasive breast cancer, with 144 (84.7%) in the control group and 153 (90%) in the experimental group. In the post-test, most participants in the control group experienced severe fatigue (136, 80%), mucositis (82, 48.2%), nausea (83, 49.1%), and vomiting (81, 47.6%). Conversely, the majority in the experimental group reported mild mucositis (110, 64.7%), nausea (92, 54.1%), and vomiting (93, 54.7%), along with moderate fatigue (116, 68.2%). Hair loss was incomplete for all participants in the control group and 115 (97.6%) participants in the experimental group. There was a significant difference between the experimental and control groups regarding fatigue (p < 0.001), insomnia (p < 0.01), anorexia (p < 0.01), mucositis (p < 0.01), nausea (p < 0.01), vomiting (p < 0.01), leukopenia (p = 0.001), neutrophil count (p < 0.01), hair loss (p < 0.05), and taste alteration (p < 0.01) during the post-test.

Conclusion

The study demonstrated that early health interventions, such as health education and progressive muscle relaxation, significantly reduced the adverse effects experienced by breast cancer patients undergoing chemotherapy. This suggests that providing supportive education and exercise training to both patients and caregivers can be beneficial in managing these side effects.

## Introduction

Breast cancer is the most frequently identified cancer among women, and is the primary cause of cancer-related deaths, after colorectal and lung cancer. In 2020, the GLOBOCAN report recorded 2.3 million cases of breast cancer, accounting for 11.7% of all cancer cases, and 6.85 lakh (6.9%) deaths globally [1]. The incidence of cancer in Kerala is increasing, with a 36% rise in new cases, according to the latest State Economic Review - 2024. The Regional Cancer Centre (RCC) in Thiruvananthapuram, known as one of the leading cancer care centers in the country, shows an increase from 11,191 cases in 2020-2021 to 15,324 cases in 2022-2023. Data from the population-based cancer registry at RCC indicates a higher prevalence of lung cancer among men and breast cancer among women [2].

The treatment for breast cancer is tailored based on the specific subtype of the disease and the extent of its spread to lymph nodes (stages II or III) or other parts of the body (stage IV) [3]. Chemotherapy is the most commonly used treatment, and with advancements in medical science, chemotherapy drugs have reduced cancer mortality and increased the life expectancy of patients. However, due to its systemic nature, chemotherapy can lead to various physical and psychosocial challenges, leading to non-compliance with the treatment [4]. Breast cancer patients undergoing chemotherapy usually receive treatment on an outpatient basis, and hence, they have to deal with treatment-related symptoms at home. Previous research highlights that cancer patients need information that is thorough, personalized, and tailored to their needs [5].

Different chemotherapy regimens may result in complications such as nausea, vomiting, hair loss, fatigue, mouth sores, digestive issues, anemia, low platelet count, susceptibility to infections, and imbalances in water and electrolytes, which can weaken patients [6,7].

A qualitative study of breast cancer patients receiving chemotherapy revealed that some patients are hesitant to seek treatment in hospitals due to a fear of hospitalization. Despite being reassured that their hair will grow back after chemotherapy, many patients remain distressed, viewing hair loss as a visible symbol of being a cancer patient undergoing treatment [8].

By practicing self-care, patients can mitigate the severity of side effects and improve their quality of life. Adequate knowledge and motivation are crucial for promoting effective self-care practices [9-13].

Guided imagery (GI) and progressive muscle relaxation (PMR) have been shown to effectively alleviate fatigue and pain, as well as enhance health-related quality of life (HRQoL) in patients receiving chemotherapy [14].

The Radiotherapy Department at Government Medical College, Thiruvananthapuram is well-organized and staffed by expert oncologists and a skilled healthcare team offering free medical services for cancer patients, including diagnosis and treatment. Annually, the department registers over 2,500 new cancer cases, with more than 1,400 patients undergoing chemotherapy each month. Breast cancer is the most prevalent type of cancer treated here. The severity of symptoms varies among these patients, often causing delays in completing chemotherapy as scheduled.

While numerous studies on different strategies to manage the side effects of patients undergoing chemotherapy exist in various countries, data specific to India, particularly Kerala, are scarce. The number of cancer cases and patients receiving chemotherapy in Kerala is rising significantly. So the investigator decided to assess the efficacy of early health intervention programs, which include health education and progressive muscle relaxation to manage the adverse effects of chemotherapy among patients with breast cancer.

## Materials and methods

Study design and setting

A randomized controlled trial design was adopted for the study. The study comprises an experimental group and a control group. The study protocol was developed in alignment with the SPIRIT (Standard Protocol Items: Recommendations for Interventional Trials) 2013 statement [15] and the CONSORT (Consolidated Standards of Reporting Trials) 2010 statement [16]. The study was conducted at the Chemotherapy Unit, Government Medical College Hospital, Thiruvananthapuram, Kerala, India from June 2020 to November 2021. The objective of the study was to assess the efficacy of early health intervention programs on the adverse effects of chemotherapy among patients with breast cancer. The participants in the control group were subjected to routine care from the chemotherapy unit. The participants in the experimental group were subjected to early health intervention programs, which included health education and PMR in addition to routine care.

Study sample and sampling

Based on the findings of an earlier study [17], the sample size for this study was calculated using the following formula:

$$N = \frac{2\sigma ^{2}(Z_{\alpha }+Z_{\beta })^{2}}{\delta ^{2}}$$

 Where σ is the standard deviation and δ is the difference in the mean (type 1 error α = 0.05 and power β = 80%). The required sample size for each group was determined to be 170, taking into account 10% attrition. Thus, the sample size for the study was 340. Female patients aged 18 years or older, who had been clinically diagnosed with breast cancer (stages I, II, or III), had an ECOG (Eastern Cooperative Oncology Group) performance status of 2 or less (indicating they were ambulatory, capable of all self-care, and spent more than half of their waking hours active), and were receiving combination chemotherapy for the first time were included in the study. Patients were excluded if they had previous chemotherapy exposure, were undergoing concurrent radiotherapy, had visual, hearing, or cognitive impairments, or had known psychiatric illnesses.

The sampling technique of the study was block randomization. A computer-generated random allocation sequence, created using Random Allocation Software, was used to produce 85 blocks with a block size of four. For allocation concealment, sequentially numbered, sealed, opaque envelopes were used. Based on this sequence, the researcher prepared 85 sealed, opaque envelopes, each containing assignments for two participants in the experimental group and two in the control group. The allocation process was designed to ensure equal numbers in each group and maintain the unpredictability of group assignments. The envelopes were organized in ascending order according to the randomization sequence. An independent person not involved in the study randomly selected the sealed envelopes and assigned participants to either the experimental or the control group. Patients with breast cancer attending the radiation oncology OPD at Medical College Hospital Trivandrum, who met the eligibility criteria, were enrolled in the study after obtaining written informed consent.

**Figure 1:**
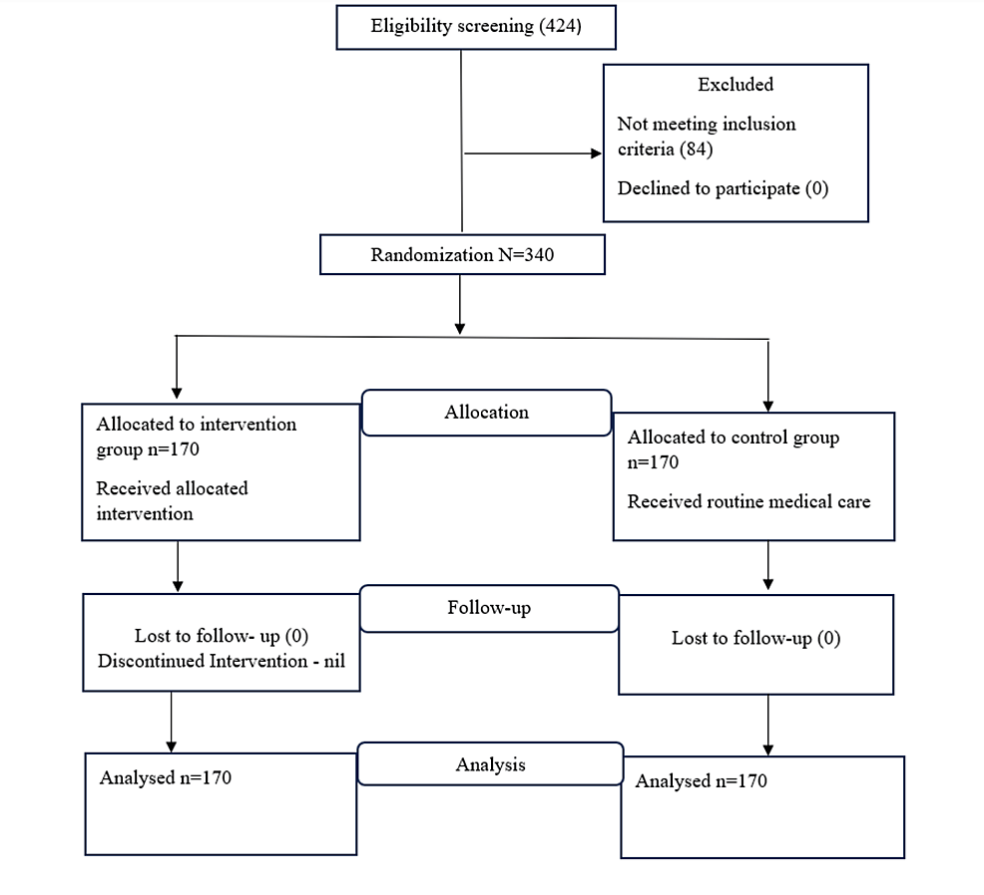
CONSORT flow chart of recruitment of participants. CONSORT: Consolidated Standards of Reporting Trials.

Figure 1 illustrates the CONSORT flow chart for the recruitment of participants in the study. A total of 424 patients were screened for eligibility, of which 340 met the criteria. These 340 participants were then equally randomized into the experimental and control groups. Notably, there were no dropouts throughout the study, and all participants were included in the final analysis.

Intervention

Participants in the control group received the standard care provided by the chemotherapy unit. In contrast, participants in the experimental group received early health intervention programs administered by the investigator in addition to the standard care. The intervention lasted for 40 minutes, which was given on the first day of chemotherapy, and included an educational session and demonstration of the PMR technique. The educational session was conducted using a lecture-discussion format, either individually or in small groups of three to four patients with PowerPoint (Microsoft Corporation, Redmond, WA) and colorful images to provide information on breast cancer chemotherapy, its adverse effects, and measures to manage these adverse effects till the end of scheduled chemotherapy. The session lasted for 20 minutes. Additionally, a pamphlet in Malayalam covering this information was distributed. Jacobson’s progressive muscle relaxation (JPMR) technique was demonstrated by the investigator to the patients and their caregivers, using an audio recording for instructions. The demonstration also lasted for 20 minutes. Participants were asked to do a return demonstration and corrections were provided as necessary. Instructions for practicing JPMR at home were also shared via an audio clip sent through WhatsApp. To reinforce the intervention, telephone follow-ups were conducted every other day during the first week, and weekly thereafter. Participants were instructed to perform JPMR exercises in the morning, evening, and whenever they experienced fatigue or nausea till the completion of chemotherapy.

Data collection tools

The data were collected through structured interviews, medical record reviews, and self-reports.

Socio-Demographic and Clinical Characteristics of the Patients

The socio-demographic data encompassed variables such as age, gender, marital status, education, occupation, place of residence, type of family, monthly family income, adverse health habits, and family history of cancer. Clinical data included details on the stage of cancer, type of breast cancer, type of chemotherapy (adjuvant or neoadjuvant), interval between cycles, comorbidities, and ECOG performance status [18]. Content validity was done by eight experts.

Common Terminology Criteria for Adverse Events, Version 3 (CTCAE v3.0)

The Common Terminology Criteria for Adverse Events (CTCAE) version 3.0, created by the US National Cancer Institute, serves to classify and grade adverse events (AEs) linked to cancer treatments [19]. This terminology is essential for reporting AEs and a severity grade was assigned to each AE term. The grading system in CTCAE version 3.0 ranges from grade 1 to grade 5, with specific clinical descriptions for each severity level: grade 1: mild AE; grade 2: moderate AE; grade 3: severe AE; grade 4: life-threatening or disabling AE; and grade 5: death related to the AE.

CTCAE v3 includes a total of 570 AEs. However, in the present study, only 16 AEs were selected based on a literature review. These include anemia, leukopenia, neutropenia, thrombocytopenia, fatigue, insomnia, hair loss, anorexia, constipation, diarrhea, oral mucositis, nausea, vomiting, taste alteration, fever, and pain (myalgia). The tool was translated to the local language, Malayalam, and back-translated to English. Since the tool is an open-access standardized one, psychometric properties were not reassessed.

Outcome measurement

Adverse effects of chemotherapy were the outcome variable of the study and were assessed with CTCAE version 3.0 in both the experimental and control groups when patients reported for their second chemotherapy session.

Ethical consideration

The trial protocol received approval from two ethics committees: the Institutional Human Ethics Committee of Government Medical College, Thiruvananthapuram (approval number: CNT/IEC/47/10/2021), where the study was conducted, and the Institutional Ethics Committee of Sri Ramachandra Institute of Higher Education and Research, Chennai (approval number: IEC-NI/19/NOV/71/88), where the study was registered. Additionally, the trial was registered with the Clinical Trials Registry-India (CTRI/2020/09/028178). The study adhered to ethical guidelines for clinical trials and followed COVID-19 protocols. Each participant received an information sheet and provided informed consent.

Procedure

Data collection was conducted from June 2020 to November 2021. Before commencing the study, formal permissions were secured from the Institutional Research Committee and the Human Ethics Committee (CNT/IEC/47/10/2021 and IEC-NI/19/NOV/71/88). Patients attending the daycare chemotherapy unit for their first cycle of chemotherapy for breast cancer were screened based on the inclusion and exclusion criteria. During the data collection period, all COVID-19 infection control protocols were strictly followed. The investigator provided a detailed explanation of the study and informed written consent was obtained from participants who met the inclusion criteria and agreed to join the study voluntarily. The consent form assured participants that their medical care at the hospital would remain unaffected by their decision to participate or withdraw, and it guaranteed the confidentiality of their data. Socio-demographic and clinical information was gathered from all participants.

Patients were assigned to experimental and control groups through block randomization. A computer-generated random number table, using a block size of four, determined the allocation. Each allocation was recorded, folded, and placed in a sealed opaque envelope. A total of 85 envelopes, numbered from 1 to 85, were prepared and randomly drawn for patient assignment. An independent department member, uninvolved in the study, carried out the allocation.

Participants in the control group received the standard care provided by the chemotherapy unit. In contrast, participants in the experimental group received early health intervention programs administered by the investigator in addition to the standard care. Both patients and their caregivers were seated comfortably in a quiet room in the chemotherapy department before the chemotherapy administration, where the intervention took place. The intervention lasted for 40 minutes and included an educational session and a demonstration of the PMR technique. An additional 10 minutes were allocated at the end of the session for answering questions, and 20 minutes were dedicated to the return demonstration of the PMR technique.

The educational session was conducted using a lecture-discussion format, either individually or in small groups of three to four patients. The session utilized PowerPoint presentations with colorful images to provide information on breast cancer chemotherapy, its adverse effects, and measures to manage these adverse effects. The session lasted 20 minutes. Additionally, a pamphlet in Malayalam covering this information was distributed. The JPMR technique was demonstrated by the investigator to the patients and their caregivers, using an audio recording for instructions. The demonstration also lasted 20 minutes. Participants were asked to do the return demonstration and corrections were provided as necessary. Instructions for practicing JPMR at home were also shared via an audio clip sent through WhatsApp. To reinforce the intervention, telephone follow-ups were conducted every other day during the first week, and weekly thereafter. Participants were instructed to perform JPMR exercises in the morning, evening, and whenever they experienced fatigue or nausea.

Participants in both groups received a CTCAE diary detailing 16 possible adverse effects of chemotherapy for breast cancer, with descriptions of their severity levels according to the CTCAE version 3. They were instructed on how to document these side effects at home following chemotherapy. Patients were asked to bring the diary with them after three weeks when they returned for their second cycle of chemotherapy. Those in the experimental group received an additional diary to record their self-care practices and PMR activities to ensure compliance. The investigator's contact number was provided for any necessary clarifications. A post-test was administered when patients returned for their second chemotherapy session, in both the experimental and the control groups.

Statistical analysis

Data were analyzed by descriptive and inferential statistics using SPSS version 25 (IBM Corp., Armonk, NY) [20]. The similarity between the groups regarding socio-demographic and clinical variables was evaluated using the chi-square test. The intervention's effectiveness was determined through the Mann-Whitney U test.

## Results

A total of 340 women with breast cancer, who were undergoing their initial chemotherapy cycle, took part in the study, with 170 participants in both the experimental and control groups. Table 1 shows that the mean age of participants was 54.7 ± 9.7 years in the control group and 52.4 ± 9 years in the experimental group. Most participants in both the control group (88, 51.8%) and the experimental group (78, 45.9%) had completed high school education. Similarly, the majority were unemployed (91 (53.5%) in the control group and 102 (60%) in the experimental group) and married (132 (77.6%) in the control group and 136 (80%) in the experimental group). Most participants in both groups belonged to the below poverty line income category (129 (75.9%) in the control group and 107 (62.9%) in the experimental group) and lived in rural areas (134 (78.8%) in the control group and 122 (71.8%) in the experimental group). Additionally, the majority of participants in both groups had nuclear families (142 (83.5%) in the control group and 141 (82.9%) in the experimental group) and no family history of cancer (127 (74.7%) in the control group and 133 (78.2%) in the experimental group). With p-values greater than 0.05 for age, education, marital status, area of residence, family type, and family history of cancer, there was no statistically significant difference between the two groups in these sociodemographic variables, indicating homogeneity among participants.

**Table 1:** Distribution of participants based on socio-demographic variables. c^2^: chi-square value. BPL: below poverty line; APL: above poverty line.

		Control		Experimental		c^2^	p
Sociodemographic variable		Number (n = 170)	Percent (%)	Number (n = 170)	Percent (%)		
Age (years)	<=50	57	33.5	72	42.4	2.95	0.229
	51-60	71	41.8	64	37.6		
	>60	42	24.7	34	20.0		
	Mean ± SD	54.7 ± 9.7		52.4 ± 9			
Education	Illiterate/primary up to 7	37	21.8	45	26.5	2.53	0.469
	High school	88	51.8	78	45.9		
	Higher secondary	26	15.3	22	12.9		
	Graduate/PG/Professional	19	11.2	25	14.7		
Occupation	Unemployed	91	53.5	102	60.0	8.45	0.038
	Unskilled worker	50	29.4	38	22.4		
	Skilled worker	9	5.3	19	11.2		
	Others	20	11.8	11	6.5		
Marital status	Married	132	77.6	136	80.0	0.28	0.595
	Others	38	22.4	34	20.0		
Monthly income of the family	BPL	129	75.9	107	62.9	6.7	0.010
	APL	41	24.1	63	37.1		
Area of residence	Rural	134	78.8	122	71.8	2.28	0.320
	Urban	16	9.4	21	12.4		
	Semi-urban	20	11.8	27	15.9		
Type of family	Nuclear	142	83.5	141	82.9	0.02	0.885
	Joint/extended	28	16.5	29	17.1		
Family history of cancer	No	127	74.7	133.0	78.2	0.59	0.443
	Yes	43	25.3	37.0	21.8		

Table 2 shows that 84 (49.4%) participants in the control group and 74 (43.5%) participants in the experiment group had stage III breast cancer. The majority in both groups had invasive breast cancer (144 (84.7%) in the control group and 153 (90%) in the experiment group). In terms of treatment, 89 (52.4%) in the control group and 103 (60.6%) in the experiment group received adjuvant chemotherapy, with most participants undergoing chemotherapy every three weeks (161 (94.7%) in the control group and 153 (90%) in the experiment group). Additionally, 123 (72.4%) participants in the control group and 112 (65.9%) in the experiment group had no comorbidities, and most participants were considered completely healthy according to the ECOG performance status (145 (85.3%) in the control group and 150 (88.2%) in the experiment group). Since the p-values of all variables are above 0.05 for cancer stage, type of cancer, chemotherapy, chemotherapy interval, comorbidities, and ECOG status, there is no statistical difference between the groups, indicating homogeneity across clinical variables.

**Table 2:** Distribution of participants based on clinical variables. c2: chi-square value; ECOG: Eastern Cooperative Oncology Group.

		Control		Experimental		c^2^	p
Clinical variables		Number (n = 170)	Percent (%)	Number (n = 170)	Percent (%)		
Stage of breast cancer	Stage I	12	7.1	21.0	12.4	3.09	0.213
	Stage II	74	43.5	75.0	44.1		
	Stage III	84	49.4	74.0	43.5		
Type of breast cancer	Invasive	144	84.7	153.0	90.0	2.16	0.340
	Infiltrating	20	11.8	13.0	7.6		
	Mucinous	6	3.5	4.0	2.4		
Type of chemotherapy	Adjuvant	89	52.4	103.0	60.6	2.35	0.126
	Neoadjuvant	81	47.6	67.0	39.4		
Interval between cycles	2 weeks	9	5.3	17.0	10.0	2.67	0.103
	3 weeks	161	94.7	153	90.0		
Comorbidities	No	123	72.4	112	65.9	1.67	0.197
	Yes	47	27.6	58	34.1		
ECOG performance status	Completely healthy	145	85.3	150	88.2	0.64	0.424
	Ambulatory, restricted/only self-care	25	14.7	20	11.8		

Table 3 shows that 136 (80%) participants in the control group experienced severe fatigue, whereas the majority of participants in the experimental group (116, 68.2%) had only a moderate level of fatigue. In terms of hair loss, most participants in both groups experienced incomplete hair loss (170 (100%) in the control group and 165 (97.6%) in the experimental group). Additionally, anemia, leukopenia, neutropenia, and thrombocytopenia were mild in the majority of patients in both groups. There was a significant difference between the experimental and control groups in terms of leukopenia (p = 0.001), neutrophil count (p < 0.01), fatigue (p < 0.001), and hair loss (p < 0.05) during the post-test. Early health intervention programs were effective in reducing adverse effects of chemotherapy, such as leukopenia, neutropenia, fatigue, and hair loss. However, no significant difference was found regarding anemia and thrombocytopenia.

**Table 3:** Efficacy of early health intervention programs on severity of adverse effects of chemotherapy among patients with breast cancer. Z#: Mann-Whitney U test; **: significant at 0.01 level; *: significant at 0.05 level.

Side effects		Control		Experimental		Z#	p
		Number (n = 170)	Percent (%)	Number (n = 170)	Percent (%)		
Anemia	Nil	1	0.6	0	0.0	0.65	0.518
	Mild	163	95.9	167	98.2		
	Moderate	6	3.5	3	1.8		
Leukopenia	Mild	135	79.4	155	91.2	3.2**	0.001
	Moderate	24	14.1	15	8.8		
	Severe	11	6.5	0	0.0		
Neutropenia	Nil	0	0.0	1	0.6	2.91**	0.004
	Mild	138	81.2	154	90.6		
	Moderate	21	12.4	15	8.8		
	Severe	11	6.5	0	0.0		
Thrombocytopenia	Nil	1	0.6	0	0.0	0.99	0.321
	Mild	168	98.8	168	98.8		
	Moderate	0	0.0	2	1.2		
	Severe	1	0.6	0	0.0		
Fatigue	Mild	0	0.0	23	13.5	13.42	<0.01
	Moderate	10	5.9	116	68.2		
	Severe	136	80.0	27	15.9		
	Disabling	24	14.1	4	2.4		
Hair loss/alopecia	Incomplete	170	100.0	165	97.6	2.01*	0.044
	Complete	0	0.0	5	2.4		

Table 4 shows that moderate severity of insomnia, anorexia, and taste alteration was observed in most of the participants in the control group (96 (56.7%), 136 (80%), and 148 (87.1%), respectively) whereas these adverse effects of chemotherapy were mild in the majority of participants in the experimental group (111 (65.3%), 95 (55.9%), and 101 (59.4%), respectively). Constipation and diarrhea were absent in the majority of participants in both groups (105 (61.8%) and 169 (99.4%) in the control group and 121 (71.2%) and 167 (98.2%) in the experimental group). Mucositis, nausea, and vomiting were severe in the majority of participants in the control group (82 (48.2%), 83 (49.1%), and 81 (47.6%), respectively) whereas these adverse effects were mild in the majority of participants in the experimental group (110 (64.7%), 92 (54.1%), and 93 (54.7%), respectively). There was a significant difference between participants in the experimental and control group with regard to insomnia (p < 0.01), anorexia (p < 0.01), constipation (p < 0.01), mucositis (p < 0.01), nausea (p < 0.01), vomiting (p < 0.01), and taste alteration (p < 0.01). Early health intervention programs were effective in reducing these side effects of chemotherapy. No significant difference was observed in the case of diarrhea.

**Table 4:** Efficacy of early health intervention programs on severity of adverse effects of chemotherapy among patients with breast cancer. Z#: Mann-Whitney U test; **: significant at 0.01 level; *: significant at 0.05 level.

Side effects		Control		Experimental		Z#	p
		Number (n = 170)	Percent (%)	Number (n = 170)	Percent (%)		
Insomnia	Nil	1	0.6	4	2.4	8.11	<0.01
	Mild	44	25.9	111	65.3		
	Moderate	96	56.5	53	31.2		
	Severe	28	16.5	2	1.2		
	Disabling	1	0.6	0	0.0		
Anorexia	Nil	0	0.0	4	2.4	11.41	<0.01
	Mild	4	2.4	95	55.9		
	Moderate	136	80.0	69	40.6		
	Severe	29	17.1	2	1.2		
	Disabling	1	0.6	0	0.0		
Constipation	Nil	105	61.8	121	71.2	3.12**	0.002
	Mild	19	11.2	45	26.5		
	Moderate	45	26.5	4	2.4		
	Severe	1	0.6	0	0.0		
Diarrhea	Nil	169	99.4	167	98.2	1.01	0.311
	Mild	1	0.6	0	0.0		
	Moderate	0	0.0	3	1.8		
Mucositis/stomatitis	Nil	1	0.6	10	5.9	13.48	<0.01
	Mild	8	4.7	110	64.7		
	Moderate	76	44.7	50	29.4		
	Severe	82	48.2	0	0.0		
	Disabling	3	1.8	0	0.0		
Nausea	Nil	0	0.0	7	4.1	12.56	<0.01
	Mild	4	2.4	92	54.1		
	Moderate	68	40.2	62	36.5		
	Severe	83	49.1	8	4.7		
	Disabling	14	8.3	1	0.6		
Vomiting	Nil	0	0.0	8	4.7	12.59	<0.01
	Mild	5	2.9	93	54.7		
	Moderate	69	40.6	61	35.9		
	Severe	81	47.6	7	4.1		
	Disabling	15	8.8	1	0.6		
Taste alteration	Nil	0	0.0	8	4.7	10.82	<0.01
	Mild	14	8.2	101	59.4		
	Moderate	148	87.1	61	35.9		
	Severe	8	4.7	0	0.0		

Table 5 shows that febrile neutropenia, pain, and numbness were absent in most of the participants, i.e., 131 (77.1%), 99 (58.2%), and 164 (96.5%), respectively, in the control group and 156 (91.8%), 113 (66.5%), and 169 (99.4%), respectively, in the experimental group. There was a significant difference in febrile neutropenia between participants in the experimental and control group (p < 0.01). Early health intervention programs were effective in reducing febrile neutropenia. No significant difference was observed in the case of pain and numbness.

**Table 5:** Efficacy of early health intervention programs on severity of adverse effects of chemotherapy among patients with breast cancer. Z#: Mann-Whitney U test; **: significant at 0.01 level; *: significant at 0.05 level.

Side effects		Control		Experimental		Z#	p
		Number (n = 170)	Percent (%)	Number (n = 170)	Percent (%)		
Febrile neutropenia	Nil	131	77.1	156	91.8	3.73**	<0.01
	Mild	3	1.8	1	0.6		
	Severe	36	21.2	13	7.6		
Pain	Nil	99	58.2	113	66.5	1.52	0.128
	Mild	57	33.5	45	26.5		
	Moderate	10	5.9	11	6.5		
	Severe	4	2.4	1	0.6		
Numbness	Nil	164	96.5	169	99.4	1.91	0.057
	Mild	6	3.5	1	0.6		

## Discussion

The present study aimed to evaluate the efficacy of early health intervention programs (health education and PMR) on the adverse effects of chemotherapy among patients with breast cancer. The study's findings indicated a significant difference between the experimental and control groups regarding adverse effects of chemotherapy, such as leukopenia (p = 0.001), neutropenia (p < 0.01), fatigue (p < 0.001), insomnia (p < 0.01), anorexia (p < 0.01), constipation (p < 0.01), mucositis (p < 0.01), nausea (p < 0.01), vomiting (p < 0.01), hair loss (p < 0.05), and taste alteration (p < 0.01) during the post-test. These results demonstrate early health intervention programs effectively managed chemotherapy-related adverse effects in breast cancer patients. Therefore, the research hypothesis, which posited that there would be a significant difference in the severity of adverse effects of chemotherapy between the experimental and control groups after the intervention, is confirmed and can be accepted.

The study's findings align with the results from a randomized clinical trial involving 60 women with breast cancer undergoing chemotherapy, which demonstrated that self-care education significantly reduced mouth sores, nausea, and vomiting [21].

A parallel-group, single-blinded, pilot, quasi-experimental trial of a psychoeducational intervention showed significant differences in fatigue, pain, and sleeping difficulty between the experimental and control groups [22]. This is also in agreement with the findings of the present study.

The current study is also backed by a randomized clinical trial that evaluated the effectiveness of GI and PMR in managing a cluster of symptoms experienced by chemotherapy patients [14]. The findings indicated that participants in the intervention group experienced significantly lower levels of fatigue and pain compared to the control group. Additionally, symptoms such as nausea, vomiting, and retching were significantly less frequent in the intervention group. While the previous study focused solely on PMR and GI, the present study incorporated both health education and PMR.

A quasi-experimental study conducted at the outpatient clinics of the Oncology Center at Mansoura University Hospital involved 80 women who had undergone mastectomy and were receiving parenteral adjuvant chemotherapy for the first time. The results demonstrated that a self-care educational program significantly reduced chemotherapy side effects such as fatigue, nausea, vomiting, mouth sores, and hair loss in the intervention group compared to the control group [23]. These findings are consistent with the results of the present study. In all the previously mentioned studies, either health education or PMR was used alone as the intervention. However, in the current study, the researcher combined both health education and PMR. As a result, significant differences were observed in a greater number of side effects compared to the other studies. In the present study, both groups were comparable in all sociodemographic variables except the family income and occupation. People from different socioeconomic statuses rely upon the Government Medical College Hospital, Thiruvananthapuram for the management of their disease conditions, especially for cancer treatment. That may be the reason for the difference between the groups in terms of occupation and family income, even though the study design was a randomized controlled trial. The strengths of the study include the study's robust design, such as the use of randomization and the control group, as well as the practical implications of the findings for managing the adverse effects of chemotherapy among patients with breast cancer.

Limitation

The study has certain limitations. Firstly, data collection was confined to a single hospital. Although the Government Medical College in Thiruvananthapuram, Kerala, serves patients from various socio-economic backgrounds, the study's scope is restricted to a specific geographic region, which affects the generalizability of the findings. Secondly, using self-reporting tools introduces some bias. It is important to recognize that while self-report measures are common and convenient, they have limitations compared to more objective methods like observation. One such limitation is that self-report measures may have lower validity and honesty, as individuals might provide inaccurate or biased information, either intentionally or unintentionally.

## Conclusions

Fatigue was the most common adverse effect of chemotherapy and was severe among the participants in the control group and moderate in the experimental group. The present study aimed to evaluate the efficacy of early health intervention programs, including health education and progressive muscle relaxation, in managing the adverse effects of chemotherapy among women with breast cancer. Results suggest that there was a significant difference in the severity of adverse effects of chemotherapy, such as fatigue, insomnia, anorexia, mucositis, nausea, vomiting, leukopenia, neutrophil count, hair loss, and taste alteration, among patients with breast cancer between experimental and control group after the intervention. The study highlights the need for early recognition of the adverse effects of chemotherapy and encouragement of patient’s involvement in their treatment and management of side effects to improve the quality of life.
